# Laparotomy, with Attachment of Ilium to Incision, in a Case of Volvulus

**Published:** 1888-06

**Authors:** J. McFadden Gaston

**Affiliations:** Atlanta, Ga., Professor of Surgery, Southern Medical College


### Southern Medical Record.

A MONTHLY JOURNAL OF PRACTICAL MEDICINE.

☞ All Communications must be addressed to R. C. WORD, M. D., Managing Editor.

"*Quicquid Præcipies Esto Brevis.*"

VOL. XVIII. ATLANTA, GA., JUNE, 1888. No. 6.

### ORIGINAL AND SELECTED ARTICLES.

### LAPAROTOMY, WITH ATTACHMENT OF ILIUM TO INCISION, IN A CASE OF VOLVULUS.

BY J. MCFADDEN GASTON, M.D., ATLANTA, GA.,
Professor of Surgery, Southern Medical College.

On the fifth of April I was requested to see a patient of Dr. C. E. Murphy, who had been suffering from obstruction of the bowels for several days, and after a caretul examination of the caes it was concluded that an operation was necessary and justifiable.

The following statement has been kindly furnished by Dr. Murphy:

I was called to see Mr. H., March 30th, about 12 o'clock. I found him suffering with severe pains in the bowels—more acute at times. He told me that at four o'clock in the morning he was awakened with a very acute pain in the right side about the region of the last rib. His mother gave him some Jamaica ginger without any relief; afterwards gave him brandy and soda, but he began to grow worse. There would be intervals of ten to fifteen minutes between the pains. His mother and father gave him a great many home preparations, but with little relief.

At 12 o'clock I was sent for and responded immediately, as I was urged to do so.

I gave him hypodermatically ¼ gr. morphia and left a mercurial purgative to be taken every three hours until bowels act freely.

A messenger was sent for me at 12 o'clock that night stating that he rested well for several hours, but was again suffering a great deal of pain. I called again. His pains this time were of a continued character, and he was vomiting up considerable amount of matter. I gave him an enema with half pint castor oil in the water. He had a small action. I again gave him hypodermatically morphia, and he slept the balance of the night. I called again early the next morning. He seemed to be suffering more from sick stomach at this time than pain.

Dr. Divine saw him with me that morning or evening. We decided to continue to give enemas and let his stomach rest for awhile; and that night I gave him 30 drops tr. opii, and a cup sweet oil, and a ½ drop croton oil. At times he was in a great deal of pain. I had decided that there was an obstruction of the bowels.

Dr. Willis Westmoreland, Sr., and Dr. Westmoreland, Jr., were called in consultation. We decided to give him opiates with hopes that rest would relieve the trouble. But finally it seemed as if opiates had but little effect. His bowels were distended so much on the left side, that I used the aspirator, drew about half gallon of undigested fecal matter. He said that he felt better and slept for several hours.

The following day he vomited a great deal of fecal matter. Strange to say during this time his temperature was never over 100°F., and most of the time normal. His pulse was a little fast when his sick spells were on, but generally normal.

Dr. Westmoreland, Jr., was called on the following day. We decided to get him in some better condition by next day, and that an operation was the last resort to relieve the obstruction.

The following day Drs. Gaston, Harris and Redwine met us at about 1 o'clock.

Mr. Ben. H. Hollingsworth, the patient, was a vigorous young man of eighteen years of age, and after using the ordinary measures for relief without effect, previous to my visit, a trocar was introduced, by Dr. Murphy, into the abdomen at two points; the puncture in the epigastric region probably entered the transverse colon and gave exit, as I was informed, to an extensive accumulation of flatus, the other, in the umbilical region, evidently penetrated the small intestines, from which more than a quart of fluid alimentary matter was discharged. The abdominal distension was evidently lessened by this process, but the tenderness upon palpation was very notable in all parts of the abdomen, and there was a sense of bogginess, without tympanitis, except in the line of the transverse colon.

The temperature was normal, and the pulse 100 beats to the minute, but there was great thirst and craving for pellets of ice constantly.

The diagnosis of obstruction of the bowels having been previously made by Dr. Murphy, it was concurred in by Drs. W. F. Westmoreland, Jr., N. O. Harris, Redwine and myself, with the conviction that nothing short of laparotomy could be relied upon for restoring the passage of the contents of the alimentary canal. We accordingly delegated Dr. Murphy to acquaint the patient and his parents with our decision, as to the prospect of a fatal termination without interference, and the probability of relief by an operation, at the same time that it would be attended with risk to life. The reply given, after understanding all the surroundings of the case, was that the proposition was accepted. After thoroughly cleansing the linia alba with soap and water, the skin was scrubbed well with a cloth saturated with spirits of turpentine. A hypodermic injection of sulph. morph., gr. ¼; sulph. atrop., gr. 1.150 was applied. The A. C. E. mixture, consisting of alcohol, f ℥i; chloroform, f ℥ii; s. ether, f ℥iii, was administered by Dr. Harris, so as to secure the full and constant anæsthetic influence. In the various steps of the operation I had the helping hand of Dr. Westmoreland, Jr., and the aid of Drs. Murphy and Redwine.

The abdomen was opened along the median line from the umbilicus to the pubes, when the site of the constriction was found to be in the right iliac region, and that extensive adhesions had already formed between the coils of the intestines and the abdominal walls in other portions of the cavity. With a view to the proper exploration of th nature of the trouble, and preparatory to the use of proper measures of relief, it was requsite to enlarge the opening of the abdomen carrying the incision through the umbilicus, to a point midway between it and the xiphoid cartilage. A portion of the small intestines enormously distended and injected was brought outside, and enveloped with towels, wrung out of hot water, when it was practicable to make out clearly that there was a volvulus of the ilium within six or eight inches of its cæcal attachment. The gut was twisted upon itself, involving a coil with a diameter of two inches, and at the lapping portions of the canal the agglutination was complete so that the entire lumen of the bowel was obliterated On either side of this occlusion there was such a disintegration of structures, that with the handling of the parts the contents of the alimentary canal escaped, and the only practicable proceeding which seemed available was ligation of the gut on the cæcal side and a division of the walls of the ilium beyond the necrosed tissue on the other side with a view to its attachment in the line of union of the abdominal walls. The idea of a resection of the implicated structure with a resection of the canal by uniting the upper and lower portions was precluded by the lower segment being bound down by adhesive inflammation, so that it was necessary to use an aneurism needle to pass the ligature around it, and the further difficulty of getting the upper segment entirely emptied of its contents by pressure. The cavity of the abdomen was washed with carbolized hot water so as to cleanse it from the deleterious extravasations of fæcal matter.

After forcing out a considerable quantity of the contents of the ilium, the end of which was carried outside of the abdominal walls, the extremity was secured with the fingers, while the abdominal incision was closed by interrupted sutures of iron dyed silk carried through all the structures, and the serous and muscular coats of the free end of the gut were caught up by several stitches which uniting it, about half way from the umbilicus to the navel, to the margins of the incised parietes. The wound was covered with antiseptic gause, and a compress placed over the open orifice of the ilium, when a broad bandage was carried around the body and fastened with pins.

In the meantime the indications of failing powers had induced Dr. Harris to suspend the anæsthetic and resort to frequent hypodermic injections of sulphuric ether with evident benefit; but we were all so impressed by the general peritonitis and the gangrenous state of the walls of ilium near the impediment, that it was, thought proper to give the family an unfavorable prognosis immediately after finishing the operation. The patient rallied from the anæsthetic, and was entirely rational for some hours with a hopeful spirit, but sank before midnight, about ten hours after the laparotomy.

Had laparotomy be n resorted to in this case within the first four days, there is good ground for the belief that it would have proved successful and the result of delay should prompt to earlier surgical steps in future cases of obstruction.

